# A Senescence-Like Cell-Cycle Arrest Occurs During Megakaryocytic Maturation: Implications for Physiological and Pathological Megakaryocytic Proliferation

**DOI:** 10.1371/journal.pbio.1000476

**Published:** 2010-09-07

**Authors:** Rodolphe Besancenot, Ronan Chaligné, Carole Tonetti, Florence Pasquier, Caroline Marty, Yann Lécluse, William Vainchenker, Stefan N. Constantinescu, Stéphane Giraudier

**Affiliations:** 1 INSERM, U790, Institut Gustave Roussy, Villejuif, France; 2 Université Paris XI, IFR54, Institut Gustave Roussy, Villejuif, France; 3 AP-HP, Université Paris XII, Laboratoire d'Hématologie, PRB Cellulothèque hématologie, Hôpital Henri Mondor, Créteil, France; 4 Ludwig Institute for Cancer Research, Brussels, Belgium; University of Virginia, United States of America

## Abstract

Thrombopoietin (TPO) via signaling through its cognate receptor MPL is a key cytokine involved in the regulation of megakaryocyte differentiation leading to platelet production. Mature megakaryocytes are polyploid cells that have arrested DNA replication and cellular proliferation but continue sustained protein synthesis. Here, we show that TPO induces cell-cycle arrest in the megakaryocytic UT7-MPL cell line by the activation of the ERK/MAPK pathway, induction of p21CIP transcription, and senescence markers through EGR1 activation. A similar senescence-like process was also detected in normal primary postmitotic megakaryocytes. In contrast, senescence was not observed in malignant megakaryocytes derived from primary myelofibrosis patients (a form of chronic myeloid hemopathy). Our data indicate that polyploid mature megakaryocytes receive signals from TPO to arrest cell proliferation and enter a senescent-like state. An escape from this physiological process may be associated with certain myeloproliferative neoplasms leading to abnormal megakaryocytic proliferation.

## Abstract 2

During normal megakaryocyte development, in response to thrombopoetin, mature cells enter a senescence-like state in which they shed platelets; this state, characterized by cell cycle arrest, is defective in malignant megakaryocytes.

## Author Summary

Megakaryocytes are huge bone marrow cells that shed platelets into the blood stream to promote clotting at sites of injury. Mature megakaryocytes differentiate from precursor cells in response to a hormone called thrombopoetin. Here, we show that as part of this normal differentiation process mature megakaryocytes enter a state called senescence in which cell division stops—a feature normally associated with cell aging and death. By studying megakaryocytes in culture, we were able to determine the biochemical pathway induced by thrombopoetin that leads to gene activation associated with senescence. We conclude that thrombopoietin acts differently at two steps in megakaryocyte differentiation: in the early stages it induces megakaryocyte proliferation, and at a latter stage it arrests the cell division cycle leading to platelet production by these cells. Interestingly, certain malignant megakaryocytes did not undergo senescence in response to thrombopoetin, which might explain the abnormal proliferation of these cancerous cells.

## Introduction

Hematopoietic stem cells (HSCs) in adults are maintained in a long term-quiescence state. On rare occasion HSCs may enter the cell cycle, and their proliferative state is usually coupled to a differentiation process regulated by both intrinsic and extrinsic factors such as cytokines (reviewed in [1]). In most somatic cells, proliferation is dependent on mitogen-activated protein kinase (MAPK) signaling, shown to be involved in the transition through the early G1 phase of the cell cycle (reviewed in [2]). Particularly, prolonged MAPK signaling is also a potent inducer of differentiation and thus links proliferation and developmental progression in somatic cells [3],[4]. However, when mature cells are produced, they are maintained in a post-mitotic state by mechanisms not yet fully understood.

Megakaryopoiesis is the hematopoietic differentiation process that leads to platelet production. The arrest of megakaryocyte proliferation is followed by ploidization resulting from endomitosis. During endomitosis, cell size and protein production per cell increase. Indeed, endomitosis corresponds to a mitosis with a failure of late cytokinesis but is still associated with DNA replication [5],[6] and transcription [7]. Usually, when megakaryocytes become 16N, the endomitosis process stops and is followed by terminal differentiation leading to cytoplasmic fragmentation and platelet shedding. The main regulator of megakaryocyte differentiation is the cytokine thrombopoietin (TPO: GeneID: 7066). TPO binds to and activates the TPO receptor (MPL: GeneID: 4352) signaling to regulate both early and late stages of differentiation [8].

Cellular senescence is a state of permanent cell-cycle arrest contributing to tissue aging and has been considered in recent years as an intrinsic barrier against tumorigenesis (reviewed in [9]–[11]). Recently, multiple secreted inflammatory cytokines, their cognate receptors, and induced transcription factors have been identified as key mediators of oncogene-induced senescence (OIS) [12]–[14]. Besides prevention of tumor outgrowth from benign lesions, other roles of OIS in non-oncogenic processes are emerging such as the fibrogenic response to acute tissue damage [15]. Senescence can be triggered by activated oncoproteins such as BRAF^E600^ or RAS^V12^ and occurs in a variety of cell types [14],[16]–[18]. OIS is accompanied by an up-regulation of CDK inhibitors, for instance p15 (GeneID: 1030) (also known as INK4B), p16 (GeneID: 1029) (also known as INK4A), and p21 (GeneID: 1026) (also known as Cip1), and is associated with an increase in the senescence-associated β-galactosidase (SA-β-Gal) activity [19]–[22]. Moreover, the senescence process is recognized as a physiologically irreversible mechanism, yet some cancer cells can escape this process [23],[24].

It has been suggested that a possible link between senescence and terminal differentiation might exist. It is well known that TPO, via binding to MPL and activation of associated tyrosine kinase JAK2 (GeneID: 371), induces a high and sustained RAS/MAPK activation in megakaryocyte precursors and mature megakaryocytes [4]. Thus we hypothesized that such a mechanism may be involved in the proliferative arrest observed in mature megakaryocytes.

## Results

**Figure 1:**
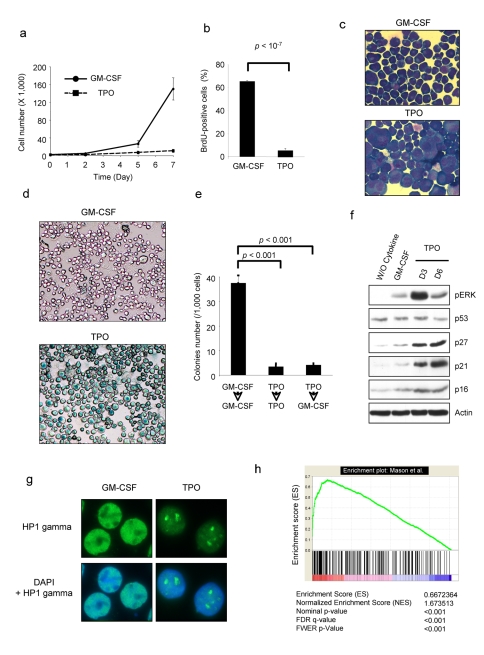
Thrombopoietin induces cellular senescence of UT711oc1 cells. (a) TPO inhibits UT711oc1 cell proliferation. UT711oc1 cells were cultured in presence of either GM-CSF or TPO. Viable cells were counted using Trypan blue exclusion. (b) TPO induces a decrease in DNA replication in UT711oc1 cells. BrdU incorporation was measured in UT711oc1 exposed for 5 d to either GM-CSF or TPO. (c) Morphological changes in TPO-stimulated UT711oc1 cells. Cells were grown for 5 d in presence of either GM-CSF or TPO and stained with May-Grünwald Giemsa. (d) SA-β-galactosidase staining in TPO-treated cells. (e) Irreversible cell-cycle arrest. UT711oc1 cells were grown for 5 d with GM-CSF or TPO and seeded in methylcellulose with GM-CSF or TPO. Cell colony numbers were determined. (f) Sustained ERK phosphorylation and p21 expression after TPO exposure. UT711oc1 cells were cultured in presence of GM-CSF or TPO and proteins were analyzed by Western blotting. (g) Formation of heterochromatin foci in UT711oc1 cells treated with TPO. Cells were grown with either GM-CSF or TPO and heterochromatin protein 1 (HP1) gamma was revealed by immunofluorescence. (h) GSEA. After 12 h cytokine starvation, UT711oc1 cells were stimulated with either GM-CSF or TPO. TPO-induced gene expression—relative to GM-CSF—was determined by micro-array analysis and compared by a Gene Set Enrichment Analysis with the molecular signature of oncogenic ras-induced senescence determined in fibroblasts by Mason et al. [29]. In histograms shown, error bars represent standard deviations of three independent experiments.

The erythro-megakaryocytic cell line UT7 does not naturally respond to TPO due to very low expression of the TPO receptor MPL but responds to GM-CSF, a hematopoietic cytokine mediating its effects on the neutrophil lineage as well as on hematopoietic progenitors, but it does not appear to have a role in basal hematopoiesis. The UT7 cell line expresses GM-CSF receptors and proliferates in response to this cytokine. We transduced the cells with a retroviral vector encoding MPL and selected a clone called UT711oc1, which expresses high level of MPL. Proliferation of UT711oc1 cells was stimulated by GM-CSF (Figure 1a). Surprisingly, in presence of TPO, UT711oc1 cells proliferated over a 2-d period before stopping (Figure 1a). This was a marked difference with the previously described UT7/MPL cells, which were selected for their growth in presence of TPO [25] but similar to other previously UT7 cell line described by Porteu et al. [26]. UT711oc1 cells could be maintained in culture for 3 wk (unpublished data). These differences are probably related to the selection processes of the clones: i.e., long term selection at a low dose of TPO for the UT7/MPL Komatsu's cells and viral transduction of mpl for Porteu's and our cells. These UT711oc1 cells were blocked in the DNA replication process (Figure 1b) and underwent morphological changes characterized by large cytoplasm and nucleus (Figure 1c). Exposure to TPO for 3 d was sufficient to render the cell unresponsive to further stimulation by GM-CSF (Figure 1e), suggesting that cell-cycle arrest was not reversible. Moreover, we did not detect any Annexin V staining in UT711oc1 cells stimulated with TPO (Figure S1a), nor were the PARP and caspase 3 cleaved (Figure S1b). On the other hand, these cells exhibited HP1gamma foci (Figure 1g), SA-β-galactosidase staining (Figure 1d), and expressed cell-cycle inhibitors such as p21CIP and p27KIP (GeneID: 102) (Figure 1f), as previously reported [27], 3 senescence-associated markers. These results were confirmed in two other MPL-overexpressing UT7 cell lines (Figure S1c). In order to confirm that TPO was able to induce senescence, other markers of senescence were analyzed. Cathepsin D (GeneID: 1509) mRNA up-regulation has been previously proposed as a senescence marker [28]. We observed a drastic increase in cathepsin D mRNA expression in TPO-exposed cells (Figure S1d). To confirm the senescence phenotype, we determined TPO-induced gene expression in 24 h TPO-exposed cells relative to that of GM-CSF-stimulated cells by micro-array analysis. The TPO-induced gene expression profile was then compared to the published molecular signature of oncogenic ras-induced senescence [29]. A gene set enrichment analysis (GSEA) comparing these two sets of genes revealed a significant enrichment for TPO-induced gene expression in senescent fibroblast genes (Figure 1h). This enrichment was highly significant for up-regulated genes. The coincident 30 most up- and down-regulated genes are depicted (Figure S2). Among the most up-regulated coincident genes, we found genes involved in inflammation (CXCL2 (GeneID: 292), PTGS2 (GeneID: 574), IL6 (GeneID: 356), IL8 (GeneID: 3576), CXCL3 (GeneID: 2921), IL1B (GeneID: 3553)) that agree with recent literature linking OIS to an interleukin-dependent inflammatory network and among the most down-regulated coincident genes appeared genes involved in DNA replication and cell proliferation. Lastly, senescent cells are able to secrete cytokines and chemokines. We analyzed TPO-exposed UT711oc1 supernatant cytokine concentrations compared to GM-CSF-exposed UT711oc1 supernatant. MCP1 (GeneID: 6347), IL-1, IL-10 (GeneID: 3586), and VEGF (GeneID: 7422) are secreted by TPO-exposed cells when compared to GM-CSF stimulated cells, cytokines previously reported to be secreted by senescent cells (Figure S1e) [30]–[33]. There are various types of senescence including the senescence induced by short telomeres and OIS. While the first mechanism is linked to long-term culturing, OIS can quickly be induced in cell lines after small GTPase RAS overstimulation. TPO has been shown to induce a high and sustained level of the RAS/MAPK signaling (Figure 1f). Altogether, these data indicate that TPO per se is able to induce a senescence process in UT711oc1 cells. We investigated the implication of MAPK activity in TPO-induced senescence.

**Figure 2:**
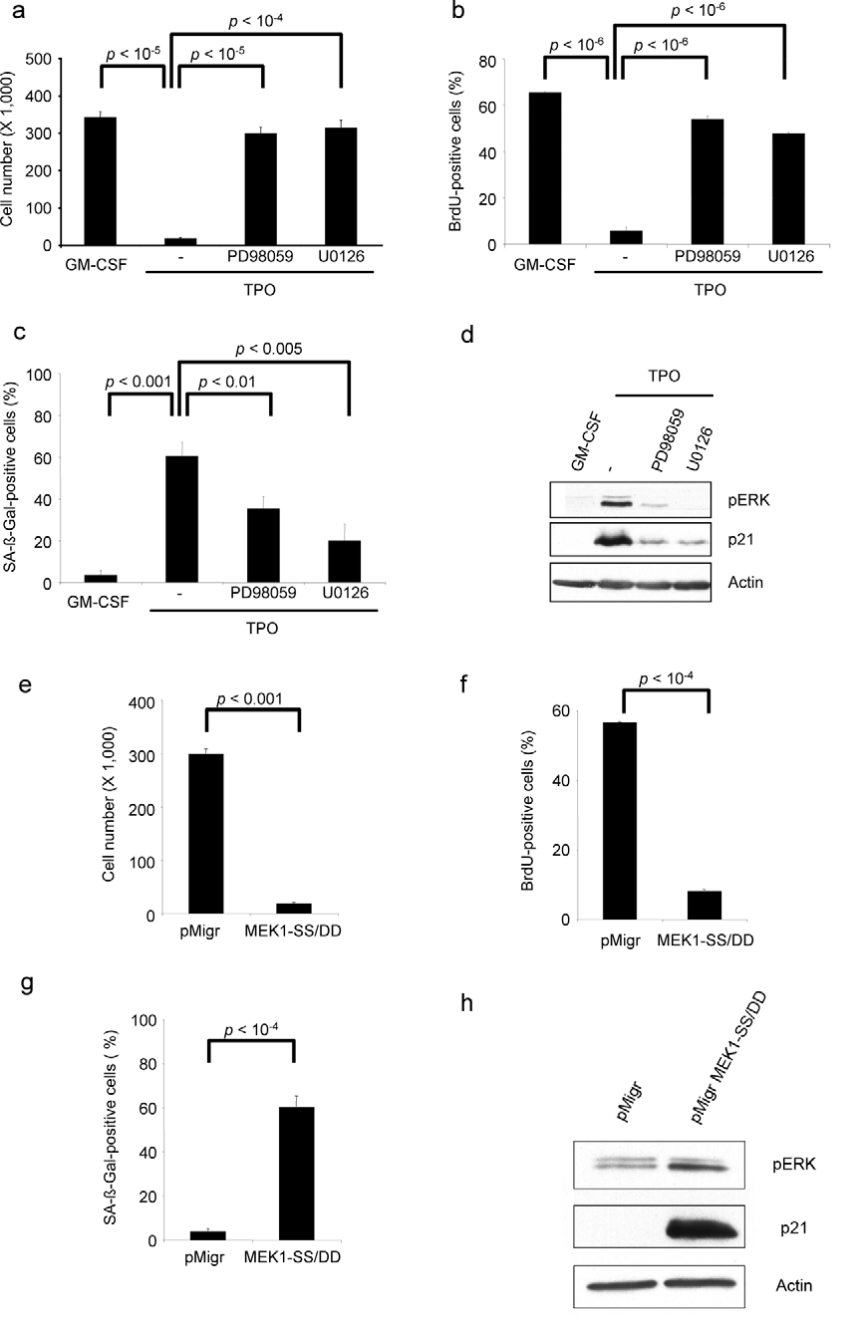
ERK regulates TPO-induced senescence. UT711oc1 cells were cultured for 5 d in presence of TPO and 10 µM of PD98059 or U0126 MAPK inhibitor. MAPK inhibitors (a) restore cell proliferation, (b) increase BrdU incorporation, (c) decrease SA-β-galactosidase activity, and (d) inhibit ERK phosphorylation and p21 protein expression. UT711oc1 cells were transduced with either empty retroviral vector (pMigr) or the vector encoding for a spontaneously active MEK (MEK1-SS/DD). MEK1-SS/DD (e) inhibits GM-CSF-dependent cell proliferation, (f) blocks BrdU incorporation, (g) induces SA-β-galactosidase staining, and (h) causes an increase in ERK phosphorylation and p21 protein expression. Error bar represents the standard deviation of three independent experiments.

In order to determine whether TPO-induced senescence was RAS/MAPK dependent, we used MAPK pathway inhibitors. RAS/MAPK inhibition either by PD98059 or U0126 reversed the TPO-induced cell proliferation arrest (Figure 2a) as previously reported [4], restored DNA replication (Figure 2b), and decreased the SA-β-galactosidase staining (Figure 2c), p21CIP level (Figures 2d and S3a), and cathepsin D expression (Figure S3b) but did not induce recurrent changes in p27KIP, p53 (GeneID: 7157), or p16INK4a expression (Figure S3c). These results illustrate that TPO-induced senescence is MAPK-dependent. In order to clearly demonstrate that the senescence phenotype was a direct consequence of sustained RAS/MAPK-pathway activation, we over-expressed a constitutively active form of MEK (MEK1-S218D/S222D) in UT711oc1 cells and cultured cells in presence of GM-CSF. We observed a proliferative arrest (Figure 2e) as previously reported [34], a DNA replication arrest (Figure 2f), an increased SA-β-galactosidase staining (Figure 2g), a rise in the p21CIP cell-cycle inhibitor transcript (Figure S3d) and protein (Figure 2h), and an up-regulation of cathepsin D mRNA (Figure S3e), but it did not affect p27KIP expression (Figure S3f). Of note, TPO stimulation or active form of MEK did not change total ERK protein expression (Figure S7). We concluded that TPO-induced senescence was a direct consequence of the ERK activation.

**Figure 3:**
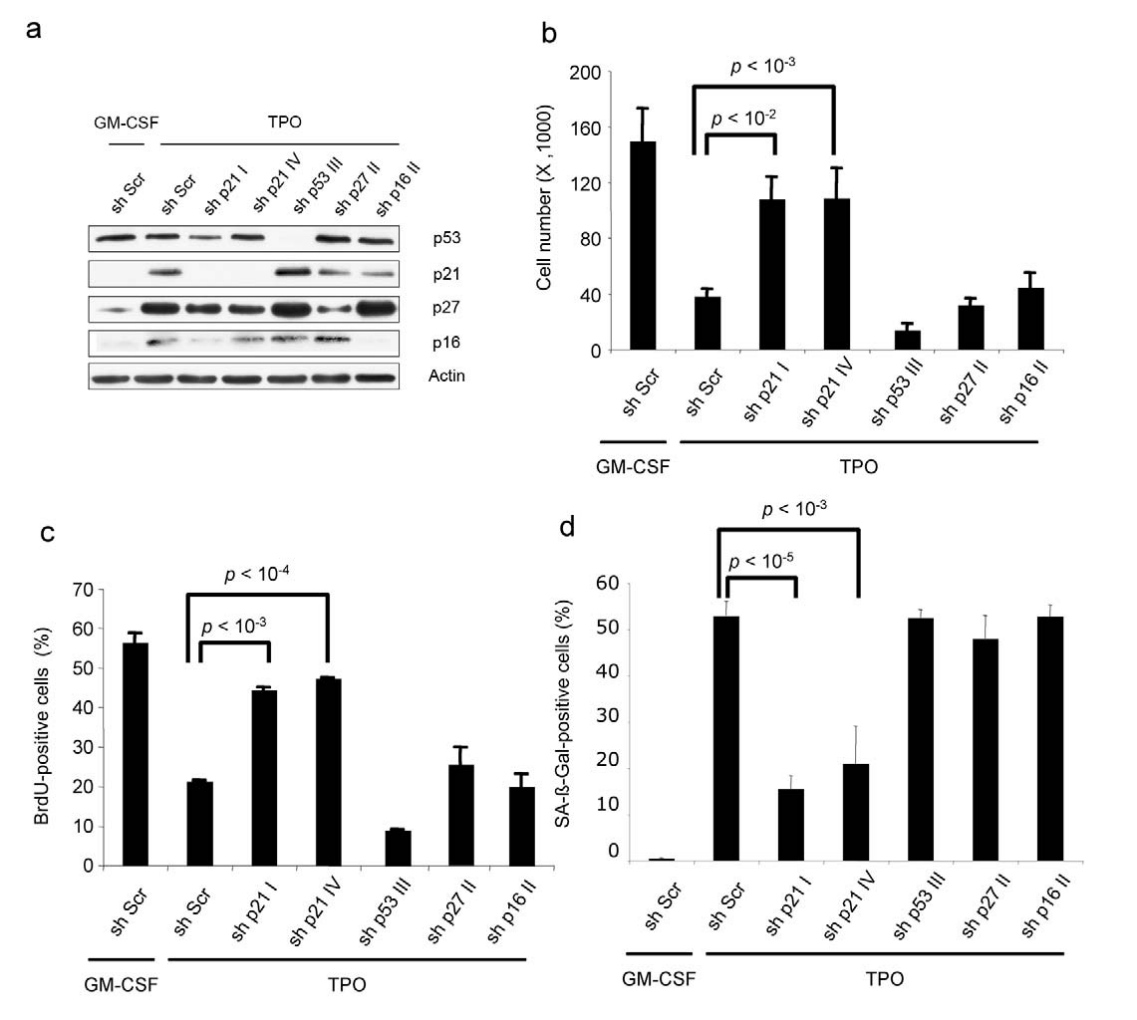
Only p21 knock-down inhibits TPO-induced UT711oc1 cell senescence phenotype. (a) shRNA lentiviral transductions of UT711oc1 cells are efficient to inhibit, respectively, p21, p27, p53, and p16 in presence of TPO. (b) TPO-stimulated proliferation of p21 shRNA over-expressing cells is restored. (c) p21 shRNA induces BrdU incorporation in presence of TPO. (d) SA-β-galactosidase staining of p21 shRNA over-expressing cells decreases. Error bar represents the standard deviation of three independent experiments.

To clarify the implication of cell-cycle inhibitors in TPO-induced senescence, we knocked down cell-cycle inhibitors (p27KIP, p21CIP, p16INK4a) and p53 in the UT711oc1 cell line using lentiviral shRNA expression. Specific shRNAs were functional at the protein level (Figure 3a). TPO induced a decrease in cell proliferation and in DNA replication in the shRNA-expressing cell lines except for p21CIP shRNAs (Figure 3b and c). Moreover, SA-β-galactosidase staining was high in all cell lines but in UT711oc1 cells expressing p21CIP shRNAs (Figure 3d). This demonstrated that TPO-induced senescence is p21CIP-dependent but p27KIP-, p16INK4a-, and p53-independent.

**Figure 4:**
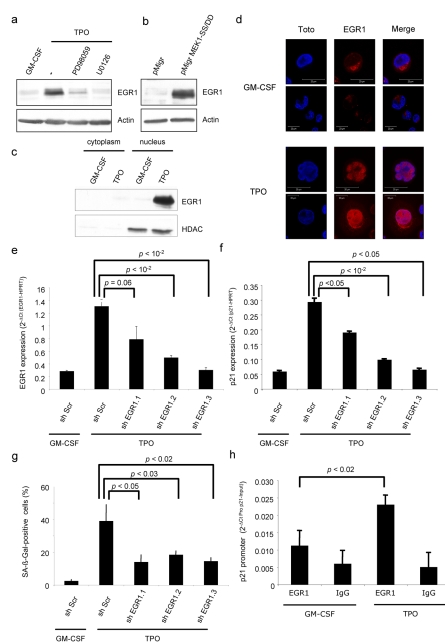
Transcription factor EGR1 increases and translocates to the nucleus in TPO-stimulated UT711oc1 cells and directly regulates p21 mRNA expression. (a) TPO-dependent EGR1 induction is inhibited by PD98056 or U0126 treatment. UT711oc1 cells were cultured for 5 d in presence of TPO and two different MAPK inhibitors. (b) EGR1 increases after MEK1-SS/DD expression in presence of GM-CSF. (c) TPO induces the translocation of EGR1 to the nucleus. Cells were grown with GM-CSF or TPO for 2 h and lysates were fractionated before being resolved by Western blotting. (d) Immunolabeling of EGR1 (in red) and nucleus (in blue) were performed and analyzed by confocal microscopy. EGR1 was present in low quantity in cytoplasm in proliferating UT711oc1 (GM-CSF), but TPO induced an increase in EGR1 labeling and translocation of the transcription factor to the nucleus. (e) EGR1 shRNA lentiviral transductions efficiently repress EGR1 mRNA expression after TPO exposure and (f) down-regulate expression of p21. (g) EGR1 knock-down inhibits the SA-β-galactosidase staining. (h) Study of chromatin immunoprecipitation (ChIP) with EGR1 antibody shows enrichment in p21 promoter after TPO exposure compared to GM-CSF. The figures represent one of three performed experiments. Error bar represents the standard deviation of three independent experiments.

We next investigated whether p21CIP was transcriptionally regulated by the MAPK pathway. We detected significant p21CIP protein expression 4 h after addition of TPO (unpublished data), suggesting the possible involvement of a transcription factor regulated by ERK. Expression of p21CIP is known to be directly regulated by several transcription factors. We proposed the early growth response protein 1 (EGR1 (GeneID: 1958)) as a candidate for TPO-induced p21CIP-dependent senescence because (i) EGR1 has been implicated in cell-cycle arrest [35], (ii) EGR1 can be activated by the RAS/MAPK pathway [36], and (iii) EGR1 regulates p21CIP transcription [37]. We first analyzed EGR1 expression in UT711oc1 cells stimulated by either GM-CSF or TPO and after ERK chemical inhibition. EGR1 was expressed in UT711oc1 cells only after TPO stimulation. Moreover, its expression was abrogated by PD98059 and U0126 inhibitors (Figure 4a). Thus, in UT71oc1 cells, EGR1 expression is regulated by TPO in a MAPK-dependent mechanism. We analyzed whether ERK signaling was the main pathway regulating EGR1 expression in our system. UT711oc1 cells infected with a constitutively active MEK (MEK1-SS/DD) and cultured in presence of GM-CSF presented an increase in EGR1 expression (Figure 4b). EGR1 is found either in the cytoplasm or the nucleus. We studied EGR1 localization after TPO stimulation using Western blotting (Figure 4c) and immunolabeling coupled with confocal microscopy (Figure 4d). Based on both these approaches we showed that EGR1 was localized in the nucleus after 2 h TPO stimulation and thus was presumably active. To examine whether EGR1 could regulate p21CIP expression following TPO stimulation, UT711oc1 cells were transduced with lentiviral vectors expressing EGR1 shRNAs. These shRNAs induced a knockdown of EGR1 at the mRNA and protein levels (Figures 4e and S4a) resulting in a decrease in expression of p21CIP after 2 h TPO stimulation (Figure 4f). Inactivation of EGR1 reversed, at least in part, the DNA replication arrest of UT711oc1 cells after TPO exposure (Figure S4b) and decreased the proportion of cells with SA-β-Galactosidase staining (Figure 4g). In order to demonstrate that EGR1 directly regulates p21CIP, chromatin immunoprecipitation ChIP analysis was performed. Briefly, after EGR1 immunoprecipitation, we detected binding to DNA sequences belonging to the p21CIP promoter. We observed an increase in EGR1 binding to p21CIP promoter after TPO stimulation (Figure 4h). Altogether these results confirmed that after TPO stimulation EGR1 was activated by the RAS/MAPK pathway, translocated to the nucleus, bound to p21CIP promoter, and activated p21CIP transcription, finally inducing senescence.

**Figure 5:**
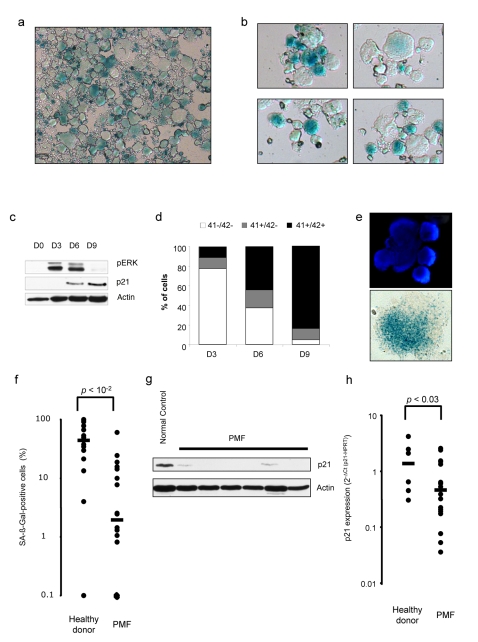
Cellular senescence is present in human and mouse mature megacaryocytes but is lacking in oncogenic megakaryocytes. (a) C57/Bl6 purified Lin^−^ were cultured in serum-free medium with 10 ng/mL TPO for megakaryocytic differentiation and stained at day 6 to reveal a SA-β-galactosidase activity. (b) Human CD34^+^ cells were cultured in serum-free medium for 10 d and SA-β-galactosidase activity was detected. (c) ERK phosphorylation status and p21 expression were analyzed by Western blotting during human megakaryopoiesis at days 0, 3, 6, and 9. (d) Levels of megakaryocyte maturation membrane markers (CD41^+^ and CD42^+^) increase during the culture of CD34^+^ cells into megacaryocytes. (e) Human megakaryocytes isolated from healthy bone marrow revealed a SA-β-galactosidase activity. One cell isolated from several present in the original image is represented. (f) Percentage of SA-β-galactosidase-positive megakaryocytes per sample were analyzed in normal and primary myelofibrosis megakaryocytes (PMFs). PMFs compared to normal megakaryocytes in culture show a significant decrease in (g) p21 protein expression and (h) p21 mRNA level. Scale bar indicates the sample median. Error bar represents the standard deviation. Each dot represents one PMF or healthy donor sample.

We studied whether TPO-induced senescence in UT711oc1 cells was a phenomenon widespread to normal megakaryocyte differentiation. Lin^−^ cells isolated from C57/Bl6 mice were cultured for 6 d with TPO and were found to display a high SA-β-galactosidase activity (Figure 5a). To extend these findings to human, CD34^+^ cells were cultured in vitro in megakaryocytic differentiation condition. We observed an increase in SA-β-galactosidase staining 10 to 16 d after the start of the culture (Figure 5b), when megakaryocytes were polyploid and initiating platelet production. Cathepsin D mRNA expression was also induced during megakaryocytic differentiation (Figure S5c). To determine whether the senescence observed in culture was associated to cell maturation, we analyzed p21CIP expression during megakaryocytic cell culture, along with CD41, CD42, and von Willebrand factor megakaryocytic markers. p21CIP expression was very low in immature cells (day 0 to day 3 of culture) and increased at day 6 and day 9. Interestingly, the peak in p21CIP expression was reached approximately 3 d after the maximum level of ERK phosphorylation (Figure 5c). There was a marked increase in megakaryocyte differentiation measured by the rising proportion of mature (CD41^+^/CD42^+^) versus progenitors (CD41^−^/CD42^−^) and immature megakaryocytes (CD41^+^/CD42^−^) at days 3, 6, and 9 (Figures 5d and S5a). CD41+ cells were positive for von Willebrand factor (Figure S5b) and this megakarycocyte differentiation was correlated with the progressive increase in cathepsin D mRNA expression (Figure S5c). Chemical inhibition of ERK activation in human megakaryocytic cultures demonstrated a decrease in p21CIP mRNA and protein expression (Figure S6a and S6b). To confirm that senescence could be defined as a physiological process happening during megakaryocyte differentiation, we sorted human mature normal CD41^+^/CD42^+^ megakaryocytes from human bone marrow aspirations and tested these cells for expression of markers of senescence. Using this approach, we confirmed that SA-β-galactosidase activity was also present in vivo in mature megakaryocytes (Figure 5e).

Cellular senescence is a permanent state of cell-cycle arrest and is emerging as an intrinsic barrier against tumorigenesis. Primary myelofibrosis (PMF) is a myeloproliferative disorder induced in 50% of cases by an acquired JAK2^V617F^ mutation leading to spontaneous kinase activation. In PMF, this mutation is present in all myeloid cells including the megakaryocytic cell line. Thus, we investigated whether such malignant megakaryocytes could escape to TPO-induced physiological senescence. Human PMF and normal CD34^+^ cells were cultured with TPO for 12 d. PMF compared to normal megakaryocytes exhibited a lower SA-β-galactosidase staining as revealed by the intensity of staining and the percentage of SA-β-galactosidase-positive cells (Figure 5f). This result suggests a defect of TPO-induced senescence in these malignant megakaryocytes. We examined whether p21CIP was down-regulated in PMF megakaryocytes and analyzed its expression in 10 d cultured normal and PMF megakaryocytes. After 10 d of culture p21CIP mRNA and protein expressions were lower in PMF than in normal megakaryocytes (Figure 5g and 5h). The down-modulation of p21CIP expression may play a role in the resistance to the physiological TPO-induced senescence process, thus leading to the increase in megakaryocyte hyper-proliferation in these myeloproliferative diseases.

## Discussion

**Figure 6:**
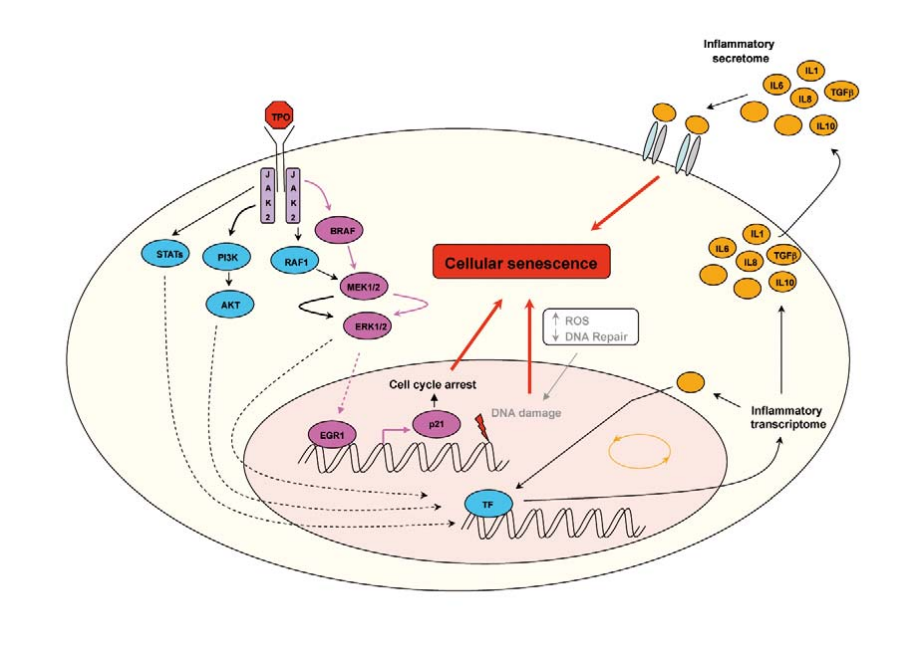
Schematic representation of transduction pathways potentially involved in TPO-induced senescence phenotype.

Hematopoietic cytokines are commonly considered as proliferative and anti-apoptotic proteins regulating blood cell production in basal or stress conditions. TPO is a specific megakaryocytic cytokine that induces proliferation and cell differentiation and regulates platelet production. In addition, in HSCs TPO induces quiescence of primitive HSCs and proliferation of the multipotent progenitors. Thus, TPO seems to have proliferative and anti-proliferative actions, depending on the cell type (reviewed in [38]). Megakaryopoiesis is a multiple stage differentiation process under the control of TPO. Megakaryocytic precursors proliferate, switch to polyploidization, and stop DNA replication before terminal differentiation leading to platelet shedding. Here, we report that UT7 cells genetically modified to over-express the TPO receptor respond to TPO by inducing senescence and that a similar process occurs in normal megakaryocytes. It was recently demonstrated that senescence is partially related to the release of inflammatory cytokines such as IL-6 and IL-8 [13],[30],[31],[33]. Upon secretion by senescent cells, these cytokines can trigger and maintain the senescence process. Our observations support the notion that TPO, a non-inflammatory cytokine, recapitulates the OIS mechanisms by inducing a high and sustained RAS/MAPK activation and consequently transcription of EGR1, leading to synthesis of the p21CIP CDKi. In addition, a recent study suggests that increased reactive oxygen species (ROS) promote megakaryopoiesis [39]. ROS accumulation driving megakaryocyte maturation could also participate in DNA damage observed in megakaryocytes (γH2AX foci observed in megakaryocytes in our laboratory) (Ali A and Debili N, unpublished data), cumulative oxidative damage lastly contributing to cellular senescence (Figure 6). This TPO-induced senescence could be a key mechanism for post-mitotic arrest without cell death, allowing these cells to respond to different stimuli with biological effects other than proliferation. In this work, we have shown the important role of p21CIP in the proliferative arrest of the UT711oc1 cells. Similarly, induction of p21CIP was also found during megakaryocyte differentiation. It is worth noting that the mouse knock-out of p21CIP does not abrogate the post-mitotic arrest during normal megakaryocyte differentiation [40], suggesting the possible involvement of other CDKis, such as p19^INK4d^ (GeneID: 1032) [41]. Furthermore, based on our results on primary cells from healthy donors and from myeloproliferative patients, it is possible that the heterochromatin changes associated with TPO-induced senescence might be more important in normal differentiation. Induction of several CDKis might occur differently in function of the differentiation stages and allow transmission of signals in a reversible manner in terminally differentiated megakaryocytes. It is possible that depending on the stage of differentiation, there will be differences in MPL and JAK2 expression levels resulting in different levels of TPO-induced ERK/MAPK signaling.

Senescence is a biological process that limits oncogenic transformation. Immature hematopoietic cells (progenitors or HSCs) have a very high proliferative capacity and linking senescence to proliferation could efficiently limit “high-risk” oncogenic processes in these cells. However, hematopoietic tissues are still the targets of oncogenic processes (i.e. leukemia and myeloproliferative disorders). Cancer cells can be defined as cells that escape the senescence process induced by an oncogene. The mechanisms leading to such an escape remain unclear. We hypothesize that in malignant megakaryocytic cells, depending on MPL signaling for their proliferation, TPO-induced senescence may be deficient due to events that counteract the MPL/MAPK/EGR1/p21CIP pathway. In favor of this hypothesis, we found that PMF megakaryocytes have lost their senescence ability in response to TPO due to a p21CIP down-expression. The entire mechanisms leading to such repression of p21CIP expression are actually not defined.

In conclusion, we describe in this report a mechanism leading to proliferation arrest in mature hematopoietic cells, the TPO-induced senescence, that is not operative in malignant megakaryocytic cells.

## Materials and Methods

### Cell Culture

UT711oc1 cells were seeded at a density of 1×10^5^ cells/mL and grown in Dulbecco's modified Eagle's medium (DMEM; Invitrogen, Cergy Pontoise, France) or in methylcellulose medium. Both media were supplemented with 10% fetal bovine serum (FBS), antibiotics (100 IU/ml penicillin and 50 mg/ml streptomycin), and GM-CSF (5 ng/mL) or recombinant human TPO (hTPO) (10 ng/mL). The MAPK inhibitors PD98059 and U0126 were used at 10 µM (Calbiochem, San Diego, CA, USA). Human CD34^+^ cells and mouse C57/Bl6 Lin^−^ cells were purified and seeded as previously described [42].

### Plasmids and Production of Retroviruses and Lentiviruses

MEK1-S218D/S222D cDNA was cloned into the bicistronic retroviral vector pMIGR-IRES-GFP. pGIPZ plasmids containing p21CIP shRNA (no. V2LHS-203118 and V2LHS-230370), p27KIP shRNA (no. V2LHS-262973), p53 shRNA (no. V2LHS-217), p16INK4a shRNA (no. V2LHS-195839), and EGR1 shRNA (no. V2LHS-262011, no. V2LHS-151347, and no. V2LHS-151348) were purchased from Open Biosystem (Thermo Scientific products, Surrey, UK). Vesicular stomatitis virus glycoprotein pseudotyped Viral particles were produced into 293EBNA or 293T cells as previously described [8],[43]. UT711oc1 cells were infected with concentrated retrovirus or lentivirus supernatants for 2 h at a multiplicity of infection of 10 and sorted by flow cytometry (FACS Vantage, BD Biosciences, Mountain View, CA) 48 h later based on eGFP expression.

### GSEA

For microarray analysis, a published data set of senescent fibroblasts (Mason et al. [29]) was used. Raw global gene expression values (from Affymetrix GeneChip) for 24 h TPO-exposed UT711oc1 cells samples and GM-CSF-exposed samples were collected. We then processed these samples with the robust multiarray analysis (RMA) algorithm using BioConductor software, version 2.3. Using the processed data, GSEA (http://www.broad.mit.edu/gsea/) was performed to look for enrichment of the Senescent-fibroblast list. Moreover, gene-array results were analyzed using Ingenuity Pathways Analysis.

### Annexin V Assay

Annexin V fluorescein isothiocyanate (FITC)-positive staining was determined by FACS analysis according to the manufacturer's recommendations (BD Pharmingen, Franklin Lakes, NJ, USA).

### Cell-Cycle and CD41/CD42 Analysis

5-Bromo-2-deoxyuridine (BrdU) labeling was performed using FITC BrdU Flow Kit (BD Biosciences, Le Pont de Claix, France) according to the manufacturer's protocol. Cells were labeled during 30 min with BrdU at 37°C in 5% CO2 atmosphere. Human cultured CD34^+^ cells were rinsed in PBS, stained for 30 min at 4°C with anti-CD41–APC (Allophycocyanin) or anti-CD42–FITC (BD Biosciences) antibodies. Control cells were incubated with an irrelevant mouse immunoglobulin G1 (IgG1) antibody. Cell samples were analyzed by FACS.

### Immunoblotting

Proteins were extracted in RIPA buffer with 1% Triton X-100 (Sigma-Aldrich, St. Louis, MO, USA) and supplemented with protease inhibitor mixture tablets (Complete, Roche Diagnostics, Meylan, France). Proteins (20–40 µg per lane) were separated on SDS–polyacrylamide gel electrophoresis and transferred to polyvinylidene difluoride (PVDF) membranes by a standard procedure. Antibodies used for immunoblotting were: Actin (Sigma-Aldrich), p21CIP (no. 2947), p16INK4a (no. 4824), PARP (no. 9542), phosphoERK (no. 9101), EGR1 (no. 4153) (Cell Signaling, Beverly, MA, USA), p27KIP (no. 510242) (BD Biosciences), p53 (no. SC-6243), and Caspase 3 (no. 7148) (Santa Cruz Biotechnology, Heidelberg, Germany). Bands were revealed using enhanced chemiluminescence (ECL, Pierce Perbio, Brebières, France). The nuclear and cytoplasmic protein separations were realized as previously described [43].

### Quantitative Real-Time PCR

Total RNA was extracted using a Trizol RNA isolation kit according to the manufacturer's protocol (Invitrogen). Transcription into cDNA was performed using random hexamers and SuperScript II reverse transcriptase (Invitrogen) according to the manufacturer's instructions. All PCR reactions used Taqman PCR Master Mix (Applied Biosystems, Foster City, CA, USA) to a final volume of 20 µl. Each cDNA sample was analyzed in triplicate in the ABI PRISM 7900 Sequence Detection System (Applied Biosystems).

### Immunofluoresence

Fixation and immunofluorescence were performed on UT711oc1 cells. The following antibodies were used: anti-HP1γ (no. 2619), anti-EGR1 (no. 4153) (cell signaling), and anti-human Von Willebrand Factor (A0082) (DakoCytomation). The appropriate secondary antibodies were conjugated with Alexa 488 or Alexa 546 (Molecular Probes-Invitrogen, Cergy-Pontoise, France). TOTO-3 iodide (Molecular Probes) or DAPI was applied for nucleus staining. Cells were examined under a Zeiss LSM 510 laser scanning microscope (Carl Zeiss, Le Pecq, France) with a 63×/1.4 numeric aperture (NA) oil objective.

### Chromatin Immunoprecipitation

Chromatin immunoprecipitations (ChIP) assays were performed using a ChIP assay kit (cell signaling) with anti-EGR1 Ab (cell signaling). These assays were performed using UT711oc1 chromatin samples. Quantification of precipitated DNA fragments was carried out on an ABI PRISM 7000 sequence detection system using Taqman probes (Eurogentec, Angers, France). Relative occupancy of the immunoprecipitated factor at a locus was calculated using the following equation: 2^(CtNegCtl−CtTarget)^, where CtNegCtl and CtTarget are mean threshold cycles of PCR done in duplicate on DNA samples from negative control ChIP (using non-immune IgG) and targeted ChIP (specific antibody).

### SA-β-Galactosidase

Detection of SA-β-galactosidase activity was performed at pH = 6 as previously described [18].

### Cytokines and Chemokines Dosage

UT711oc1 cells were cultured with either GM-CSF or TPO for 6 d and secreted factor concentrations were measured in the supernatants using Procarta Protein Profiling Assays according to the manufacturer's protocol.

### Statistical Test

We performed the *t* test for all experiment. *p* indicates value of Student's *t* test.

## Supporting Information

Figure S1 **Thrombopoietin induces cellular senescence of UT711oc1 and not apoptosis.** (a) TPO and GM-CSF induce similar levels of apoptosis in UT711oc1 cells. Cells were treated with TPO for 3 and 6 d and compared to GM-CSF-cultured cells for Annexin V labeling. (b) No difference in PARP and Caspase 3 cleavage between TPO- and GM-CSF-treated cells. UT711oc1 were cultured in presence of GM-CSF or TPO as indicated and PARP and Caspase 3 proteins were analyzed by Western blotting. The “W/O Cytokine” condition represents 24 h cytokine starvation and serves as a positive control for PARP and Caspase 3 cleavage. (c) Two other over-expressing MPL UT7 cell lines, called clone 5.1 and clone 86, were generated and SA-β-galactosidase activity was evaluated after 5 d of exposure to TPO or GM-CSF. (d) TPO up-regulates cathepsin D mRNA. Cells were exposed to TPO or GM-CSF for 3 to 6 d and cathepsin D mRNA expression determined by Taqman. (e) UT711oc1 supernantants of 48 h TPO-exposed and GM-CSF-exposed cells (1 million cells/ml) were collected and cytokine concentrations were measured (in pg/ml). Three independent experiments for each apoptosis assay were performed.
(1.55 MB TIF)

Figure S2 **Top coincident genes when TPO-induced gene expression profile was compared to the molecular signature of oncogenic ras-induced senescence established in fibroblasts by Mason et al. [29].** (a) Top coincident up-regulated genes and their involvement in “growth, proliferation, and apoptosis”; “inflammation”; or “DNA replication, recombination, and repair,” when total coincident genes were analyzed using Ingenuity Pathways Analysis. (b) Top coincident down-regulated genes and their involvement in “growth, proliferation, and apoptosis,” “inflammation,” or “DNA replication, recombination, and repair.”
(1.96 MB TIF)

Figure S3 **p21 mRNA expression is up-regulated by TPO via the RAS/MAPK pathway.** (a) p21 mRNA expression in UT711oc1 cells. Cells were treated with GM-CSF or TPO in presence of PD98056 and U0126 inhibitors and assayed for gene expression by Taqman. (b) Cathepsin D mRNA expression in UT711oc1 cells. Cells were treated with GM-CSF or TPO in presence of PD98056 and U0126 inhibitors and assayed for gene expression by Taqman. (c) p53, p16, and p27 protein expressions were analyzed by Western blotting. Cells were treated in the same conditions as in (a). (d) p21 mRNA expression is up-regulated in UT711oc1 cells over-expressing a spontaneous active form of MEK (MEK1 SS/DD). (e) Cathepsin D mRNA expression is up-regulated in UT711oc1 cells over-expressing a spontaneous active form of MEK (MEK1 SS/DD). (f) p27 protein expression stays unchanged in presence of active MEK. Error bar represents the standard deviation of three independent experiments.
(1.18 MB TIF)

Figure S4 **EGR1 shRNAs are functional and re-induce cell proliferation after TPO exposure.** (a) EGR1 shRNAs inhibit EGR1 protein expression after TPO exposure. (b) BrdU incorporation at 5 d of culture shows a significant (but partial) increase in DNA replication with TPO when cells express EGR1 shRNAs. Error bar represents the standard deviation. We performed three independent experiments.
(0.18 MB TIF)

Figure S5 **Cathepsin D and megakaryocytic differentiation markers in normal human megakaryocytes.** (a) Proportion of CD41 and CD42 expressing cells during the megakaryocytic culture. (b) Co-expression of vWF and CD41 markers in megakaryocytes after 12 d of cell culture. (c) Cathepsin D mRNA expression during megakaryocytic differentiation process of human cytapheresis CD34+ cells.
(0.66 MB TIF)

Figure S6 **Inhibition of ERK decreases p21 expression in human megakaryocytes.** Human megakaryocytes were cultured for 10 d in presence of TPO and 10 µM of U0126 MAPK inhibitor. U0126 inhibited ERK phosphorylation (b) and p21 protein (b) and mRNA expression (a). We performed three independent experiments.
(0.11 MB TIF)

Figure S7 **TPO and MEK1SS/DD expression induces ERK phosphorylation without any changes in total ERK expression.** (a) UT711oc1 cells were treated with GM-CSF or TPO ± PD98059 or UO126 for 2 d. ERK phosphorylation and total ERK expression were then evaluated by WB. (b) UT711oc1 cells transduced with either empty retroviral vector (pMigr) or the vector encoding for a spontaneously active MEK (MEK1-SS/DD) were cultured in presence of GM-CSF for 3 to 6 d. ERK phosphorylation and total ERK expression were then evaluated by WB.
(0.16 MB TIF)

## Abbreviations

GSEA: gene set enrichment analysis
HSCs: hematopoietic stem cell(s)
MAPK: mitogen-activated protein kinase
MK(s): megakaryocyte(s)
MPD(s): myeloproliferative disorder(s)
PMF: primary myelofibrosis
ROS: reactive oxygen species
TPO: thrombopoietin
