# Supplementary figures and images for: A Senescence-Like Cell-Cycle Arrest Occurs During Megakaryocytic Maturation: Implications for Physiological and Pathological Megakaryocytic Proliferation
Source: PLoS Biol. 2010 Sep 7;8(9):e1000476. doi: 10.1371/journal.pbio.1000476 (PMC2935456; doi:10.1371/journal.pbio.1000476)

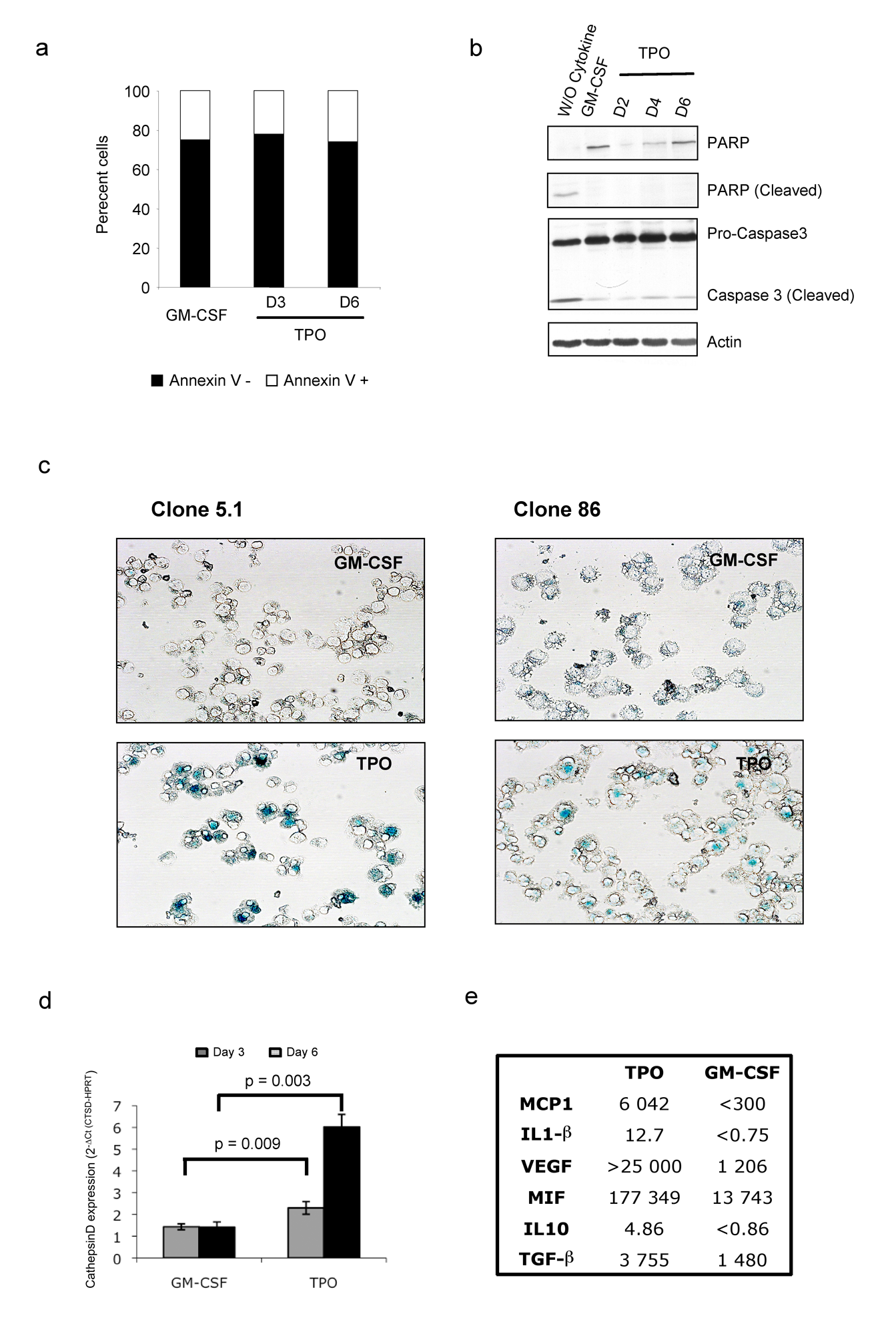

Supplement: Figure S1 — Thrombopoietin induces cellular senescence of UT711oc1 and not apoptosis. (a) TPO and GM-CSF induce similar levels of apoptosis in UT711oc1 cells. Cells were treated with TPO for 3 and 6 d and compared to GM-CSF-cultured cells for Annexin V labeling. (b) No difference in PARP and Caspase 3 cleavage between TPO- and GM-CSF-treated cells. UT711oc1 were cultured in presence of GM-CSF or TPO as indicated and PARP and Caspase 3 proteins were analyzed by Western blotting. The “W/O Cytokine” condition represents 24 h cytokine starvation and serves as a positive control for PARP and Caspase 3 cleavage. (c) Two other over-expressing MPL UT7 cell lines, called clone 5.1 and clone 86, were generated and SA-β-galactosidase activity was evaluated after 5 d of exposure to TPO or GM-CSF. (d) TPO up-regulates cathepsin D mRNA. Cells were exposed to TPO or GM-CSF for 3 to 6 d and cathepsin D mRNA expression determined by Taqman. (e) UT711oc1 supernantants of 48 h TPO-exposed and GM-CSF-exposed cells (1 million cells/ml) were collected and cytokine concentrations were measured (in pg/ml). Three independent experiments for each apoptosis assay were performed. (1.55 MB TIF) [file pbio.1000476.s001.tif]

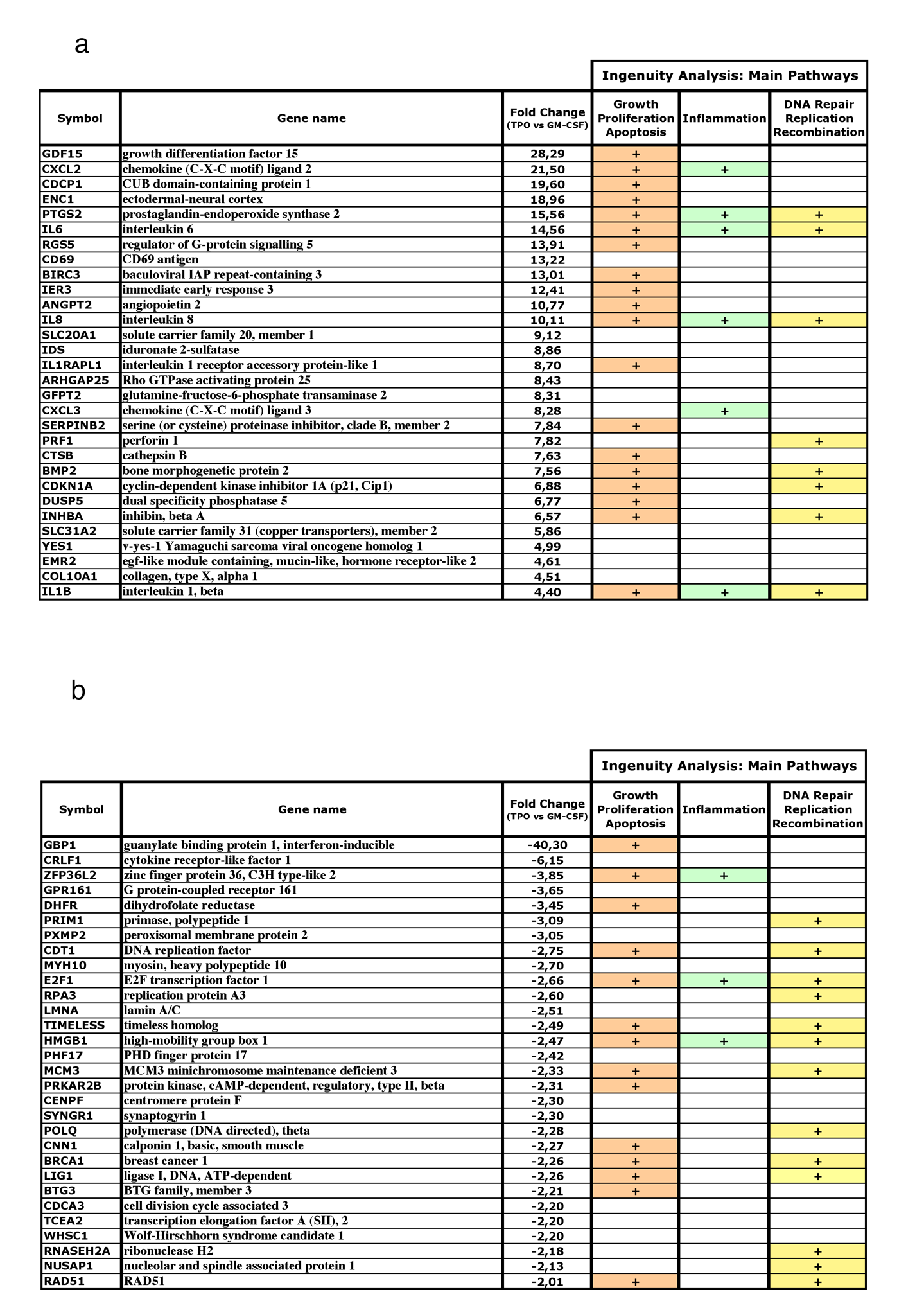

Supplement: Figure S2 — Top coincident genes when TPO-induced gene expression profile was compared to the molecular signature of oncogenic ras-induced senescence established in fibroblasts by Mason et al. [29]. (a) Top coincident up-regulated genes and their involvement in “growth, proliferation, and apoptosis”; “inflammation”; or “DNA replication, recombination, and repair,” when total coincident genes were analyzed using Ingenuity Pathways Analysis. (b) Top coincident down-regulated genes and their involvement in “growth, proliferation, and apoptosis,” “inflammation,” or “DNA replication, recombination, and repair.” (1.96 MB TIF) [file pbio.1000476.s002.tif]

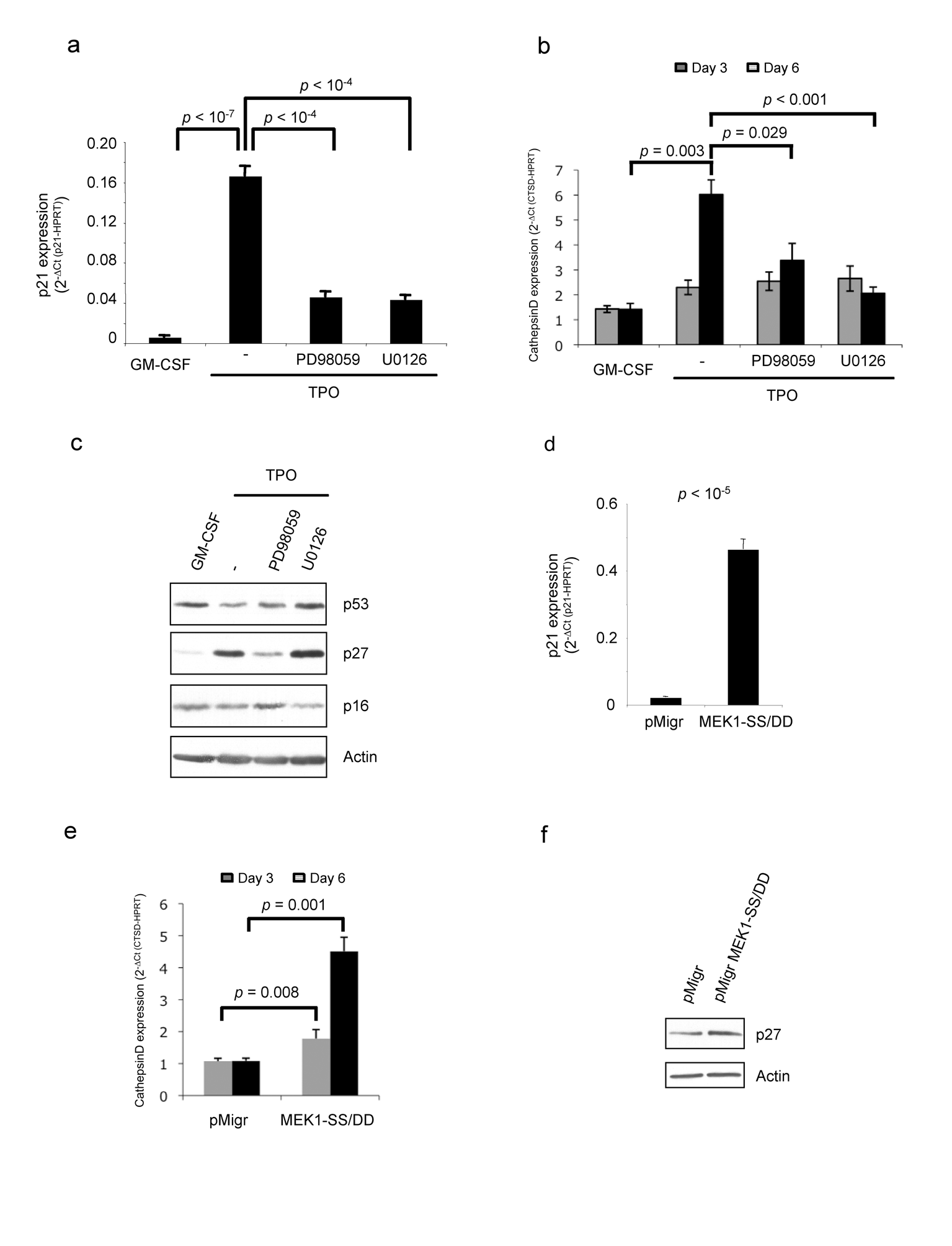

Supplement: Figure S3 — p21 mRNA expression is up-regulated by TPO via the RAS/MAPK pathway. (a) p21 mRNA expression in UT711oc1 cells. Cells were treated with GM-CSF or TPO in presence of PD98056 and U0126 inhibitors and assayed for gene expression by Taqman. (b) Cathepsin D mRNA expression in UT711oc1 cells. Cells were treated with GM-CSF or TPO in presence of PD98056 and U0126 inhibitors and assayed for gene expression by Taqman. (c) p53, p16, and p27 protein expressions were analyzed by Western blotting. Cells were treated in the same conditions as in (a). (d) p21 mRNA expression is up-regulated in UT711oc1 cells over-expressing a spontaneous active form of MEK (MEK1 SS/DD). (e) Cathepsin D mRNA expression is up-regulated in UT711oc1 cells over-expressing a spontaneous active form of MEK (MEK1 SS/DD). (f) p27 protein expression stays unchanged in presence of active MEK. Error bar represents the standard deviation of three independent experiments. (1.18 MB TIF) [file pbio.1000476.s003.tif]

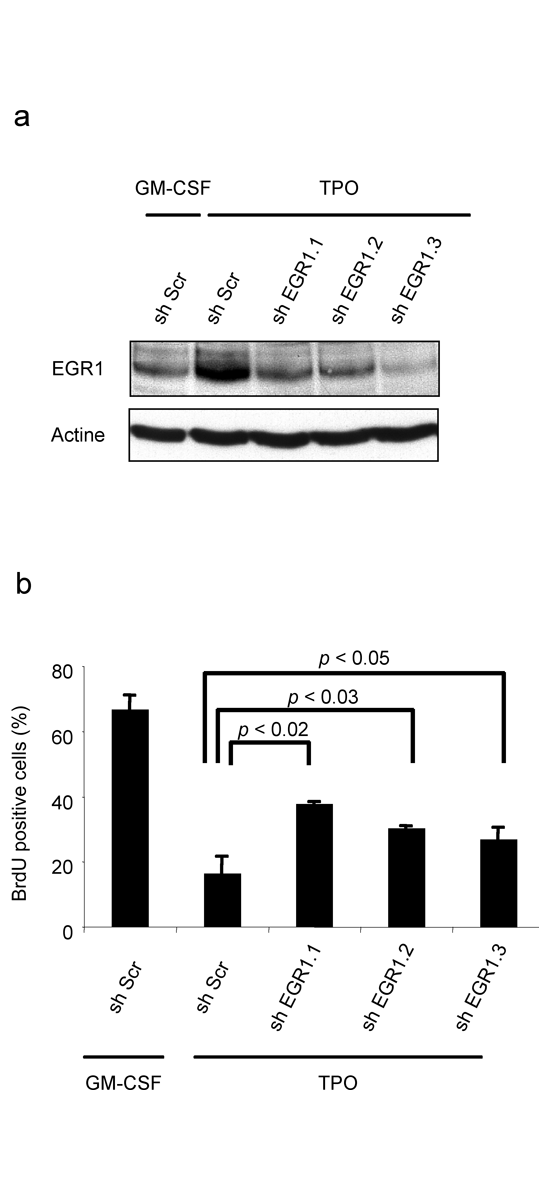

Supplement: Figure S4 — EGR1 shRNAs are functional and re-induce cell proliferation after TPO exposure. (a) EGR1 shRNAs inhibit EGR1 protein expression after TPO exposure. (b) BrdU incorporation at 5 d of culture shows a significant (but partial) increase in DNA replication with TPO when cells express EGR1 shRNAs. Error bar represents the standard deviation. We performed three independent experiments. (0.18 MB TIF) [file pbio.1000476.s004.tif]

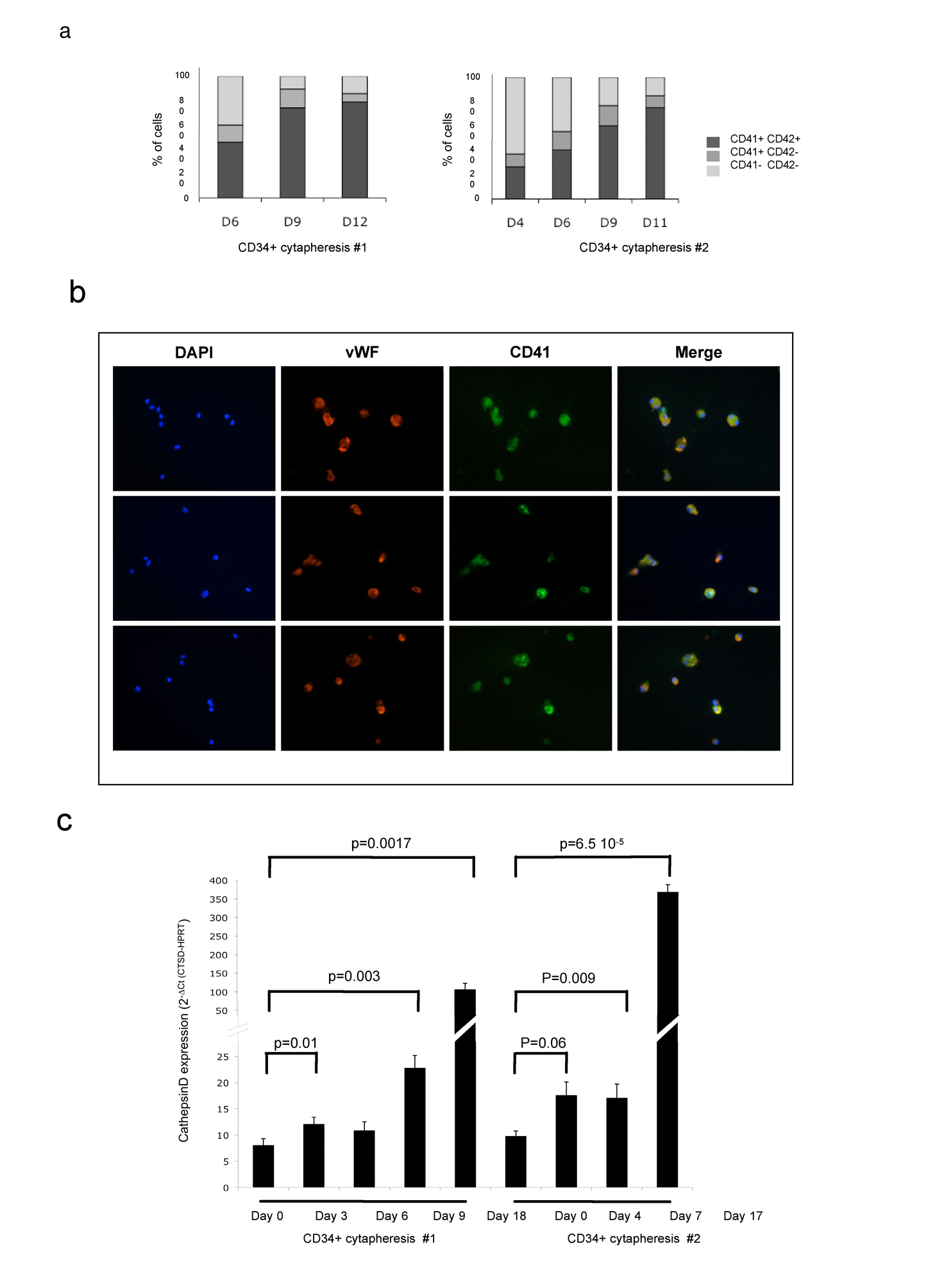

Supplement: Figure S5 — Cathepsin D and megakaryocytic differentiation markers in normal human megakaryocytes. (a) Proportion of CD41 and CD42 expressing cells during the megakaryocytic culture. (b) Co-expression of vWF and CD41 markers in megakaryocytes after 12 d of cell culture. (c) Cathepsin D mRNA expression during megakaryocytic differentiation process of human cytapheresis CD34+ cells. (0.66 MB TIF) [file pbio.1000476.s005.tif]

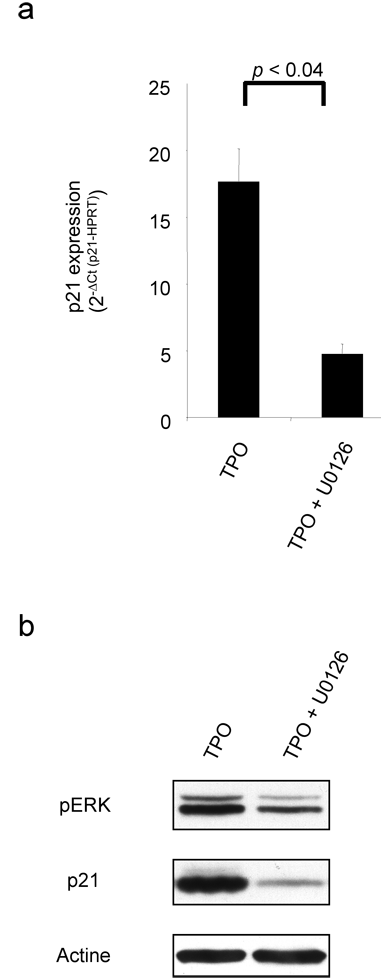

Supplement: Figure S6 — Inhibition of ERK decreases p21 expression in human megakaryocytes. Human megakaryocytes were cultured for 10 d in presence of TPO and 10 µM of U0126 MAPK inhibitor. U0126 inhibited ERK phosphorylation (b) and p21 protein (b) and mRNA expression (a). We performed three independent experiments. (0.11 MB TIF) [file pbio.1000476.s006.tif]

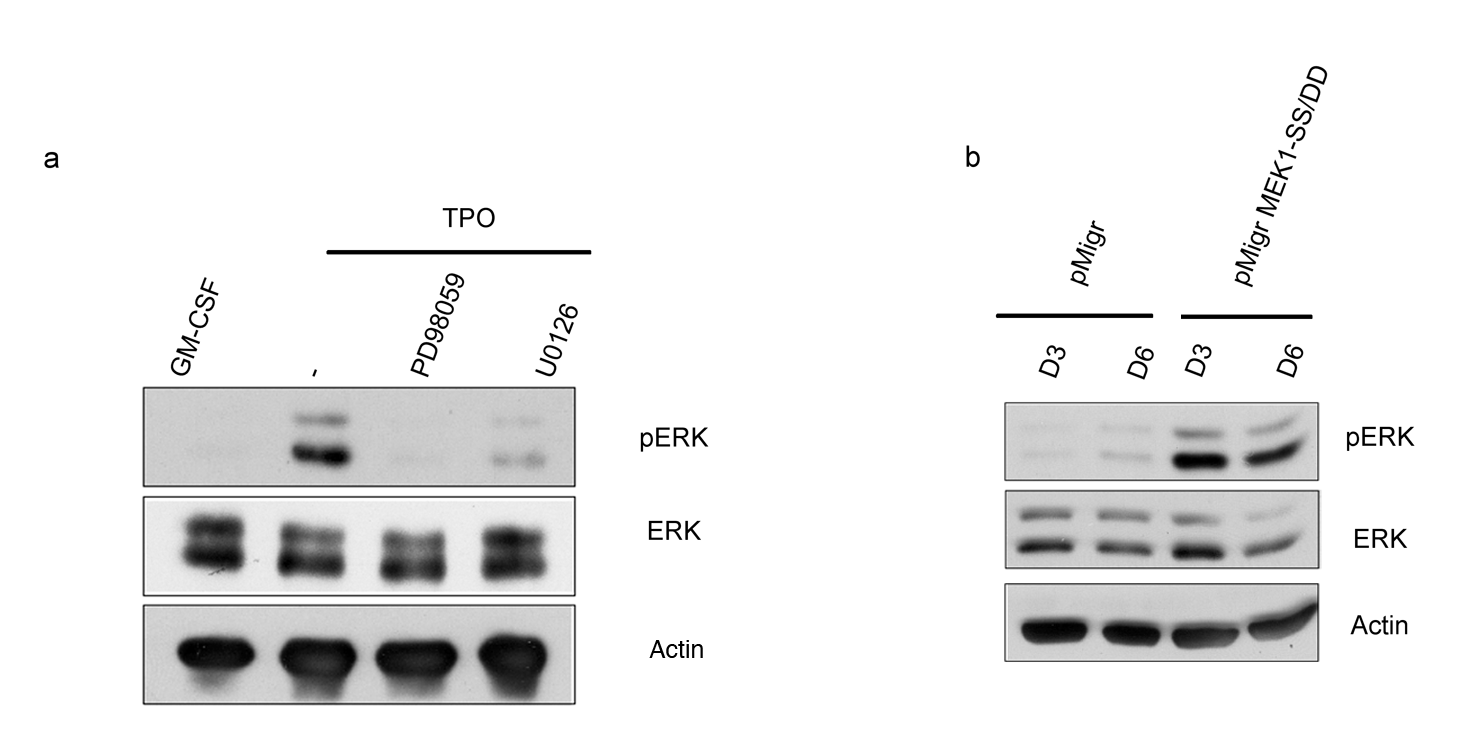

Supplement: Figure S7 — TPO and MEK1SS/DD expression induces ERK phosphorylation without any changes in total ERK expression. (a) UT711oc1 cells were treated with GM-CSF or TPO ± PD98059 or UO126 for 2 d. ERK phosphorylation and total ERK expression were then evaluated by WB. (b) UT711oc1 cells transduced with either empty retroviral vector (pMigr) or the vector encoding for a spontaneously active MEK (MEK1-SS/DD) were cultured in presence of GM-CSF for 3 to 6 d. ERK phosphorylation and total ERK expression were then evaluated by WB. (0.16 MB TIF) [file pbio.1000476.s007.tif]
